# Ethnic and Racial Inequalities in Notified Cases of Tuberculosis in Brazil

**DOI:** 10.1371/journal.pone.0154658

**Published:** 2016-05-13

**Authors:** Paulo Victor de Sousa Viana, Maria Jacirema Ferreira Gonçalves, Paulo Cesar Basta

**Affiliations:** 1 Centro de Referência Professor Hélio Fraga, Fundação Oswaldo Cruz, Rio de Janeiro, Rio de Janeiro, Brazil; 2 Instituto Leônidas e Maria Deane, Fundação Oswaldo Cruz, Manaus, Amazonas, Brazil; 3 Escola de Enfermagem de Manaus, Universidade Federal do Amazonas, Manaus, Amazonas, Brazil; 4 Escola Nacional de Saúde Pública Sergio Arouca, Fundação Oswaldo Cruz, Rio de Janeiro, Rio de Janeiro, Brazil; The Ohio State University, UNITED STATES

## Abstract

**Objective:**

This study analysed clinical and sociodemographic aspects and follow-up for notified cases of tuberculosis (TB) and explored inequalities in incidence rates and outcome by colour or race and the geographic macro-regions of Brazil.

**Methods:**

This paper reports the results of a population-based descriptive epidemiological study of all notified cases of TB in Brazil during the period from 01/01/2008 to 31/12/2011. We analysed sociodemographic and clinical variables according to colour or race (white, black, Asian, mixed, and indigenous) and geographic macro-regions of the country (North, Northeast, Central-West, South, and Southeast).

**Results:**

During the study period, the average incidence of TB in Brazil was 36.7 cases per 100,000 inhabitants, with the highest rates occurring in the North and Southeast regions. The analysis of TB notifications by colour or race revealed that the indigenous population presented the highest incidence rates in all macro-regions except the South, where higher rates were reported in black patients. ‘Cured’ was the most frequently reported treatment outcome for all skin colour categories. The highest cure rate occurred among the indigenous population (76.8%), while the lowest cure rate occurred among the black population (70.7%). Rates of treatment default were highest among blacks (10.5%) and lowest among the indigenous population (6.9%). However, the fatality rate was similar across race categories, varying between 2.8% and 3.8% for whites and the indigenous population, respectively. The lowest cure rates were observed when follow-up was inadequate (58.3%), and the highest was observed when the follow-up was classified as excellent (96.8%).

**Conclusions:**

This study revealed that—apart from the heterogeneous distribution of TB among the Brazilian macro-regions—ethnic-racial inequalities exist in terms of clinical-epidemiological characteristics and incidence rates as well as follow-up for cases undergoing treatment. The highest rates of TB occurred among the indigenous people.

## Introduction

Tuberculosis (TB) has been recognized as an important cause of illness and death in humans for thousands of years [1]. Even in the 21st century, the disease remains a contemporary health problem, especially in countries with high social inequality [2].

TB is endemic in Brazil. On average, there are 70,000 new cases and 4,000 deaths each year. In the last two decades, Brazil has made advances in controlling the disease, especially in mortality rate reduction and in expanding its direct treatment coverage in urban centres [3]. Most recently, the introduction of rapid molecular testing has simplified the process of diagnosing new cases. Despite these important advances, the disease remains one of the principal public health challenges in Brazil.

The main challenges in controlling the disease are high rates of treatment default, structural flaws in municipal control programmes, HIV co-infections, and—more recently—the emergence of drug-resistant bacterial strains, an association with diabetes mellitus, and the concentration of cases among vulnerable populations, mainly the homeless, migrant, refugee, prisoner, healthcare professional, and indigenous populations [2,4–7].

Research interest in TB among indigenous populations in Brazil has increased in recent years, especially after the official Manual of Recommendations for the Control of Tuberculosis (Manual de Recomendações para o controle da Tuberculose) highlighted their plight [8]. We now know that TB disproportionately affects indigenous populations; the incidence rates registered in these populations are systematically higher than those in the general population at both the regional and national levels [9–13].

Recently, the Ministry of Health, through the National Program for Tuberculosis Control (Programa Nacional de Controle da Tuberculose (PNCT)), ratified this situation by reporting that, based on colour and race category data from the System for Notifiable Disease (Sistema de Informação de Agravos de Notificações (SINAN)), the average TB incidence for patients who declared themselves 'indigenous' was 93.5/100,000 inhabitants in 2011, a figure nearly three times higher than the average registered for the entire population of the country during the same year [14]. Despite this knowledge, epidemiological studies aiming to explore and understand the roles of ethnic and racial inequality among risk factors for TB are scarce in Brazil [15,16].

In this context, the aim of this study is to analyse clinical and sociodemographic aspects and follow-up for reported cases of TB in Brazil and to explore inequalities in incidence rates and outcome according to colour or race and geographic macro-region.

## Methods

### Area, population, and study design

We carried out a descriptive population-based epidemiological study covering the entire national area. We analysed all notified cases of TB registered in the SINAN in Brazilian territory.

In Brazil, TB is a disease for which notification is compulsory and treatment is offered free of charge when a case is reported to the SINAN. Thus, all cases known to the Brazilian public health system (Sistema Único de Saúde (SUS)) during the study period were included.

### Variables and criteria for inclusion and exclusion

The following variables were analysed: i) sociodemographic factors, including sex, age group (0–9 years, 10–19 years, 20–44 years and ≥45 years), colour or race (white, black, Asian, mixed, and indigenous), educational level (illiterate, 1–8 years, 9–11 years, 12 or more years, unknown, and not applicable), origin (urban, rural, urban/rural and unknown), and macro-region (North, Northeast, Central-West, South, and Southeast); ii) clinical factors, including the clinical form of TB (pulmonary, extrapulmonary, both pulmonary and extrapulmonary) and the examinations used for diagnosis (bacilloscopy and sputum culture, thorax radiography, tuberculin test, HIV serology test); iii) follow-up variables, including control bacilloscopies in the 2nd, 4th, and 6th months of treatment, as well as data on supervised treatment and testing of contacts; and iv) treatment outcome, including cure, treatment default, death (from TB), and incidences of drug-resistant TB.

In Brazil, urban/rural areas are called “peri-urban” and are considered as areas located beyond the city suburbs—a space where rural and urban activities are mixed. Peri-urban areas make it difficult to determine the physical and social limits of urban and rural spaces.

We use the terminology formally adopted by the Brazilian Institute of Geography and Statistics (IBGE), which acknowledges the difficulty of measuring people’s race or ethnicity. Therefore, IBGE surveys instead ask people their phenotype, as expressed by skin colour. This allows health professionals and researchers to use the same terminology to facilitate data acquisition and question standardization and improves the ability to make data comparisons. Ethnicity is based on a person’s self-declared skin colour and includes the following categories: 'white', 'black', 'mixed', 'Asian', and 'indigenous' [17]. In Brazil, the “mixed” ethnic category is called “Pardo,” which means a mixture of European, black and Amerindian.

This study included all cases of TB where the variable 'type of entry' was designated as either 'new case' or 'unknown' and excluded cases where the ‘type of entry’ involved a diagnostic error and the variable 'outcome' was classified as 'change of diagnosis'. The study period covered the period from 01/01/2008 to 31/12/2011.

The SINAN database, which includes notified TB cases in Brazil, was obtained from the PNCT in August 2013. Duplicate cases had already been removed.

### Analysis

Following the recommendations of the PNCT [8], the incidence rate was calculated as a fraction using the total number of cases where 'type of entry' was classified as either 'new case' or 'unknown' as the numerator, while the denominator consisted of the total Brazilian population at risk during the calendar years 2008, 2009, 2010, and 2011, multiplied by 100,000.

To define the populations used to calculate the incidence rates, yearly population estimates by macro-region and for each colour/race category during the intercensus period were employed, using geometric interpolation for 2008 and 2009 and extrapolation for 2011, based on the demographic census results from 2000 and 2010 [18].

For the analysis of the operational indicators related to the follow-up of TB cases, we used an empirical classification system developed by Orellana et al. [19], which considers three recommendations from the 'III Diretrizes para TB' by the Brazilian Society for Pulmonology and Phthisiology (Sociedade Brasileira de Pneumologia e Tisiologia (SBPT)) [20]: 1) whether the notified case underwent control bacilloscopies at the 2nd, 4th, and 6th months of treatment; 2) whether examinations of contacts were registered; and 3) whether the treatment occurred under supervision. Cases where none or only one of the three recommendations were performed were classified as 'inadequate follow-up'. Cases where two of the recommendations were performed were classified as 'poor follow-up'. Cases where all three recommendations were performed were classified as 'good follow-up'. Finally, cases where treatment occurred under supervision, examinations of contacts were registered and at least two bacilloscopies were accomplished were classified as 'excellent follow-up'.

All analyses conducted within this investigation were stratified to elucidate possible inequalities between the colour/race categories and the geographic macro-regions of Brazil.

The data were structured in electronic spreadsheets using *Microsoft Excel* 2010 (Microsoft Corp., Redmond, WA, USA), and analyses were carried out using the *Statistical Package for the Social Sciences*, *version* 20.0 (SPSS Inc., Chicago, IL, USA).

### Ethical considerations

The study was approved by the Ethical Research Committee of the Escola Nacional de Saúde Pública/FIOCRUZ (CEP/ENSP), protocol number: CAAE: 14643713.0.0000.5240.

## Results

During the study period, 278,674 new cases of TB in Brazil were reported to the SINAN. The number of new case notifications among the macro-regions was highest in the Southeast region (129,573 cases, 46.5%) and lowest in the Central-West region (12,367 cases, 4.4%).

During the study period, compliance in the completion of the field “colour or race” increased: the proportion of 'unknown' entries decreased from 16.2% in 2008 to 7.8% in 2011. Among the notified cases that presented valid information on this variable, an increase of 13.5% was observed in the proportion of the population referring to themselves as 'mixed' colour or race—from 36.6% in 2008 to 42.3% in 2011. Entries of ‘indigenous’ represented 1.1% of the notifications in 2008 and 1.2% in 2011 (Table 1).

**Table 1:** Annual Distribution of Notified New Cases of Tuberculosis in Brazil, by Macro-Region and Colour or Race, in the Period 2008–2011. Source: Sinan-TB/MS. Note: *In Brazil, the Mixed race category is called Pardo, which means a mixture of European, Black and Amerindian.

Variables		2008		2009		2010		2011	
Macro-Region	Colour or Race	N	%	N	%	N	%	N	%
**North**	White	1135	18.1	1051	15.4	885	13.2	932	13.3
	Black	418	6.7	445	6.5	446	6.7	458	6.5
	Asian	58	0.9	47	0.7	45	0.7	36	0.5
	Mixed*	4269	68.0	4803	70.6	4902	73.3	5122	73.2
	Indigenous	271	4.3	270	4.0	242	3.6	256	3.7
	Unknown	130	2.1	188	2.8	167	2.5	197	2.8
	**Subtotal**	**6281**	**100.0**	**6804**	**100.0**	**6687**	**100.0**	**7001**	**100.0**
**Northeast**	White	3463	18.3	3551	18.2	3127	17.0	3176	16.6
	Black	2732	14.4	2729	14.0	2468	13.4	2562	13.4
	Asian	312	1.6	308	1.6	203	1.1	215	1.1
	Mixed	10965	57.8	11400	58.3	11047	60.0	11700	61.3
	Indigenous	127	0.7	131	0.7	107	0.6	129	0.7
	Unknown	1371	7.2	1443	7.4	1451	7.9	1318	6.9
	**Subtotal**	**18970**	**100.0**	**19562**	**100.0**	**18403**	**100.0**	**19100**	**100.0**
**Central-West**	White	997	32.3	940	31.1	901	29.2	887	27.9
	Black	362	11.7	340	11.3	321	10.4	344	10.8
	Asian	49	1.6	47	1.6	49	1.6	30	0.9
	Mixed	1347	43.6	1387	46.0	1459	47.4	1499	47.1
	Indigenous	203	6.6	213	7.1	207	6.7	261	8.2
	Unknown	130	4.2	91	3.0	144	4.7	159	5.0
	**Subtotal**	**3088**	**100.0**	**3018**	**100.0**	**3081**	**100.0**	**3180**	**100.0**
**Southeast**	White	11110	34.0	12613	39.3	13203	41.4	13474	41.0
	Black	3886	11.9	4375	13.6	4401	13.8	4699	14.3
	Asian	270	0.8	265	0.8	264	0.8	279	0.8
	Mixed	7840	24.0	9177	28.6	9519	29.8	10609	32.3
	Indigenous	138	0.4	127	0.4	144	0.5	186	0.6
	Unknown	9407	28.8	5558	17.3	4382	13.7	3647	11.1
	**Subtotal**	**32651**	**100.0**	**32115**	**100.0**	**31913**	**100.0**	**32894**	**100.0**
**South**	White	6166	75.3	6382	74.3	6296	74.4	6318	72.7
	Black	892	10.9	1007	11.7	986	11.7	1012	11.7
	Asian	40	0.5	41	0.5	47	0.6	46	0.5
	Mixed	867	10.6	891	10.4	873	10.3	1039	12.0
	Indigenous	27	0.3	39	0.5	48	0.6	32	0.4
	Unknown	202	2.5	224	2.6	213	2.5	238	2.7
	**Subtotal**	**8194**	**100.0**	**8584**	**100.0**	**8463**	**100.0**	**8685**	**100.0**
**Brazil**	White	22871	33.1	24537	35.0	24412	35.6	24787	35.0
	Black	8290	12.0	8896	12.7	8622	12.6	9075	12.8
	Asian	729	1.1	708	1.0	608	0.9	606	0.9
	Mixed	25288	36.6	27658	39.5	27800	40.6	29969	42.3
	Indigenous	766	1.1	780	1.1	748	1.1	864	1.2
	Unknown	11240	16.2	7504	10.7	6357	9.3	5559	7.8
	**Total**	**69184**	**100.0**	**70083**	**100.0**	**68547**	**100.0**	**70860**	**100.0**

**Fig 1:**
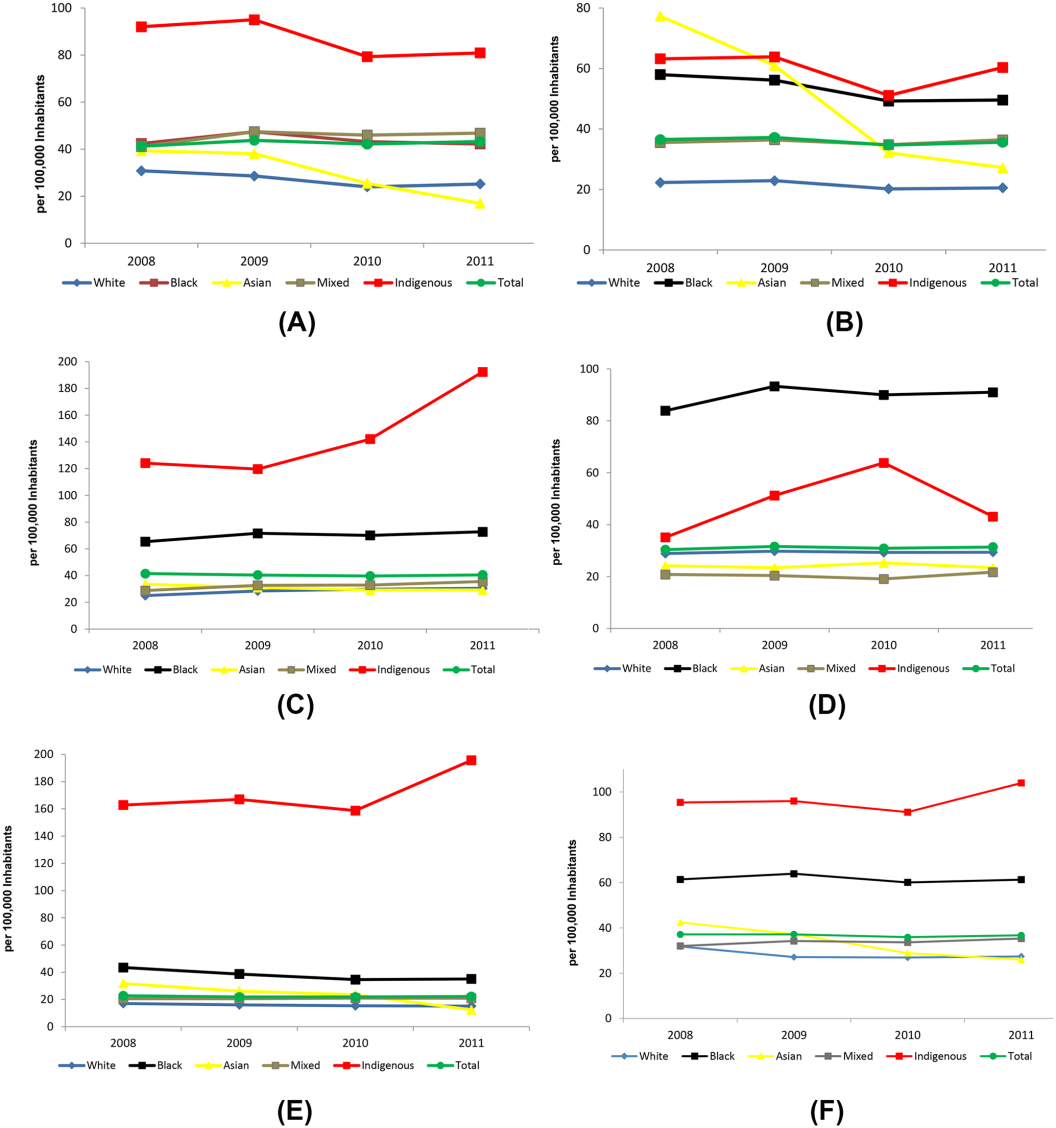
Incidence rate (per 100,000) of all clinical forms of tuberculosis, according to race and country macro-region, Brazil 2008–2011. Note: Each letter represents one macro-region from the total area of Brazil: (A) North; (B) Northeast; (C) Southeast; (D) South; (E) Central-West; and (F) Brazil.

The average incidence rate of all forms of TB in Brazil was 36.7 cases per 100,000 people from 2008–2011. The incidence rate decreased slightly during the study years, from 37.1/100,000 in 2008 to 36.7/100,000 in 2011. However, we found a heterogeneous distribution between the macro-regions. The North region presented the highest incidence rates of the country between 2009 (43.8/100,000) and 2011 (43.2/100,000); however, in 2008, the Southeast region had the highest incidence rate (41.5/100,000). In contrast, the lowest incidence rates were observed in the Central-West region, with 22.8/100,000 in 2008 and 22.2/100,000 in 2011 (Fig 1).

The indigenous population showed the highest incidence rates in all macro-regions except the South, where higher incidence rates were found in the black population: 83.9/100,000 in 2008 and 91/100,000 in 2011. The increase of 18.8% in the incidence rate for the indigenous population in the Central-West region between 2008 and 2011 stood out, increasing from 162.8/100,000 to 195.7/100,000 during that period. The lowest incidence rates were observed in individuals self-classified as 'white' in all the macro-regions except the South, where the 'mixed' population presented the lowest rates (Fig 1).

TB notifications were more frequent among men (65.7%) than among women, with an average national ratio of 1.9:1.0 (Table 2). The ratio between men and women varied according to the colour/race category and was highest among the black population (2.0:1.0) and lowest in the indigenous population (1.5:1.0).

**Table 2:** Distribution of Notified New Cases of Tuberculosis in Brazil, by Sex, Age Group, Educational Level, and Residence Area, According to Colour or Race, Brazil, 2008–2011. Source: Sinan-TB/MS. Note: * In Brazil, the Mixed race category called Pardo, which means a mixture of European, Black and Amerindian.

Colour or race—Brazil														
2008–2011	White		Black		Asian		Mixed*		Indigenous		Unknown		Total	
Sex	Cases	%	Cases	%	Cases	%	Cases	%	Cases	%	Cases	%	Cases	%
Male	62629	64.8	23347	66.9	1681	63.4	72635	65.6	1890	59.8	21020	68.6	183202	65.7
Female	33977	35.2	11534	33.1	970	36.6	38077	34.4	1260	39.9	9637	31.4	95455	34.3
Unknown	1	0.0	2	0.0	0	0.0	3	0.0	8	0.0	3	0.0	17	0.0
**Age group (years)**														
45 +	37635	39.0	12749	36.5	1053	39.7	38562	34.8	870	27.5	10696	34.9	101565	36.4
20 to 44	50667	52.4	19162	54.9	1354	51.1	60570	54.7	1568	49.7	17004	55.5	150325	53.9
10 to 19	6456	6.7	2388	6.8	207	7.8	9099	8.2	412	13.0	2220	7.2	20782	7.5
0 to 9	1849	1.9	584	1.7	37	1.4	2484	2.2	308	9.8	740	2.4	6002	2.2
**Educational level (years of schooling)**														
Illiterate	2788	2.9	2528	7.2	99	3.7	6954	6.3	505	16.0	222	0.7	13096	4.7
1 to 8	34152	35.4	15984	45.8	978	36.9	48751	44.0	1146	36.3	2098	6.8	103109	37.0
9 to 11	14030	14.5	4210	12.1	293	11.1	16172	14.6	204	6.5	809	2.6	35718	12.8
12 or +	8218	8.5	815	2.3	210	7.9	3361	3.0	45	1.4	961	3.1	13610	4.9
Unknown	35235	36.5	10662	30.6	1035	39.0	33052	29.9	1023	32.4	25755	84.0	106762	38.3
Not applicable	2184	2.3	684	2.0	36	1.4	2425	2.2	235	7.4	815	2.7	6379	2.3
**Residence area**														
Urban	62746	64.9	25560	73.3	1799	67.9	81065	73.2	850	26.9	12681	41.4	184701	66.3
Rural	4889	5.1	2796	8.0	198	7.5	11214	10.1	1713	54.2	889	2.9	21699	7.8
Urban/Rural	479	0.5	250	0.7	13	0.5	796	0.7	32	1.0	105	0.3	1675	0.6
Unknown	28493	29.5	6277	18.0	641	24.2	17640	15.9	563	17.8	16985	55.4	70599	25.3
**Total**	**96607**	**100.0**	**34883**	**100.0**	**2651**	**100.0**	**110715**	**100.0**	**3158**	**100.0**	**30660**	**100.0**	**278674**	**100.0**

Individuals aged 20–44 years comprised the majority of notified cases in all colour/race categories. Nevertheless, there were noteworthy variations, particularly in the numbers of notified cases for the age group 0–9 years. Indigenous children in this age group presented the highest rate (9.8%). In contrast, all other colour/race groups presented rates below 2.5%. Counterintuitively, the indigenous population had the lowest rate of TB cases in the age group 45 years or more (27.5%) (Table 2).

More than a third (38.3%) of the notifications included no data for the variable 'education', with similar percentages of missing information across all colour/race categories; the lowest rate was found in the 'mixed' population (29.9%) and the highest in the 'Asian' population (39.0%). The indigenous population had the highest proportion of illiterates (16.0%), followed by the black population (7.2%) (Table 2).

Regarding the variable 'area of residence' in the country as a whole, 66.3% of the notified TB cases occurred among people living in urban areas. This was true for all colour/race categories except ‘indigenous’, of whom 54.2% lived in rural areas (Table 2).

The first and second sputum smear samples for diagnosis were not performed in approximately one quarter of the cases; the lowest frequency occurred in diagnoses of white patients (23%). Sputum culture was not performed in 77.3% of cases, with the lowest percentage of non-compliance among the indigenous population (68.4%) and the highest among the 'mixed' population (80.3%) (Table 3).

**Table 3:** Clinical-Epidemiological Characteristics and of Follow-up of New Cases of TB, by Clinical Form, Diagnostic Tests (Bacilloscopy, X-ray, Culture, Tuberculin Test, and HIV test), and Type of Entry, According to Colour or Race, Brazil, 2008–2011. Source: Sinan-TB/MS. BK = Bacilloscopy; RX = Radiography; TT = Tuberculin test; MDR-TB = Multidrug-resistant tuberculosis. Note: * In Brazil, the Mixed race category is called *Pardo*, which means a mixture of European, Black and Amerindian

2008–2011	White		Black		Asian		Mixed*		Indigenous		Unknown		Total	
BK 1st sample	Cases	%	Cases	%	Cases	%	Cases	%	Cases	%	Cases	%	Cases	%
Positive	49144	50.9	19874	57.0	1516	57.2	61917	55.9	1499	47.5	17100	55.8	151050	54.2
Negative	25232	26.1	8464	24.3	596	22.5	27633	25.0	1074	34.0	6952	22.7	69951	25.1
Not performed	22231	23.0	6545	18.8	539	20.3	21165	19.1	585	18.5	6608	21.6	57673	20.7
**BK 2nd sample**														
Positive	25522	26.4	12012	34.4	833	31.4	39490	35.7	893	28.3	5168	16.9	83918	30.1
Negative	15854	16.4	5950	17.1	371	14.0	20321	18.4	822	26.0	2571	8.4	45889	16.5
Not performed	25412	26.3	9729	27.9	720	27.2	30179	27.3	820	26.0	5704	18.6	72564	26.0
Unknown	29819	30.9	7192	20.6	727	27.4	20725	18.7	623	19.7	17217	56.2	76303	27.4
**Sputum culture**														
Positive	12366	12.8	3588	10.3	240	9.1	9856	8.9	589	18.7	4160	13.6	30799	11.1
Negative	8105	8.4	2155	6.2	171	6.5	6212	5.6	266	8.4	2388	7.8	19297	6.9
Ongoing	3982	4.1	1801	5.2	141	5.3	5713	5.2	143	4.5	1413	4.6	13193	4.7
Not performed	72154	74.7	27339	78.4	2099	79.2	88934	80.3	2160	68.4	22699	74.0	215385	77.3
**RX of thorax**														
Suspect	77341	80.1	28241	81.0	2115	79.8	87519	79.0	2426	76.8	22818	74.4	220460	79.1
Normal	6417	6.6	1787	5.1	139	5.2	5265	4.8	182	5.8	1622	5.3	15412	5.5
Other pathology	815	0.8	218	0.6	13	0.5	728	0.7	19	0.6	168	0.5	1961	0.7
Not performed	9725	10.1	4002	11.5	311	11.7	15341	13.9	484	15.3	4146	13.5	34009	12.2
Unknown	2309	2.4	635	1.8	73	2.8	1862	1.7	47	1.5	1906	6.2	6832	2.5
**TT**														
No reaction	5357	5.5	1503	4.3	108	4.1	6122	5.5	222	7.0	834	2.7	14146	5.1
Weak reaction	2040	2.1	636	1.8	46	1.7	2237	2.0	86	2.7	300	1.0	5345	1.9
Strong reaction	11153	11.5	3982	11.4	287	10.8	12811	11.6	554	17.5	1709	5.6	30496	10.9
Not performed	49760	51.5	22537	64.6	1563	59.0	71561	64.6	1752	55.5	10930	35.6	158103	56.7
Unknown	28297	29.3	6225	17.8	647	24.4	17984	16.2	544	17.2	16887	55.1	70584	25.3
**Clinical form**														
Pulmonary	76496	79.2	29304	84.0	2203	83.1	93534	84.5	2735	86.6	24995	81.5	229267	82.3
Extrapulmonary	16412	17.0	4446	12.7	384	14.5	14052	12.7	334	10.6	4592	15.0	40220	14.4
Both pulmonary and extrapulmonary	3679	3.8	1130	3.2	63	2.4	3116	2.8	89	2.8	1051	3.4	9128	3.3
Unknown	20	0.0	3	0.0	1	0.0	13	0.0	0	0.0	22	0.1	59	0.0
**HIV test**														
Positive	10035	10.4	3473	10.0	148	5.6	9008	8.1	93	2.9	3034	9.9	25791	9.3
Negative	52876	54.7	15610	44.7	1233	46.5	49513	44.7	1560	49.4	15531	50.7	136323	48.9
Ongoing	6335	6.6	3642	10.4	259	9.8	11031	10.0	232	7.3	2030	6.6	23529	8.4
Not performed	27361	28.3	12158	34.9	1011	38.1	41163	37.2	1273	40.3	10065	32.8	93031	33.4
**BK 2nd month**														
Positive	5216	5.4	1778	5.1	127	4.8	5608	5.1	134	4.2	1402	4.6	14265	5.1
Negative	25354	26.2	9402	27.0	707	26.7	33430	30.2	1161	36.8	6775	22.1	76829	27.6
Not performed	45526	47.1	15460	44.3	1046	39.5	43748	39.5	1289	40.8	12134	39.6	119203	42.8
Unknown	20511	21.2	8243	23.6	771	29.1	27929	25.2	574	18.2	10349	33.8	68377	24.5
**BK 4th month**														
Positive	1100	1.1	307	0.9	26	1.0	982	0.9	25	0.8	299	1.0	2739	1.0
Negative	23989	24.8	8828	25.3	652	24.6	31503	28.5	1056	33.4	6384	20.8	72412	26.0
Not performed	47605	49.3	16031	46.0	1086	41.0	45415	41.0	1346	42.6	12218	39.8	123701	44.4
Unknown	23913	24.8	9717	27.9	887	33.5	32815	29.6	731	23.1	11759	38.4	79822	28.6
**BK 6th month**														
Positive	548	0.6	161	0.5	19	0.7	561	0.5	13	0.4	130	0.4	1432	0.5
Negative	25829	26.7	9615	27.6	742	28.0	34848	31.5	1229	38.9	6763	22.1	79026	28.4
Not performed	42871	44.4	13991	40.1	913	34.4	38242	34.5	1082	34.3	10663	34.8	107762	38.7
Unknown	27359	28.3	11116	31.9	977	36.9	37064	33.5	834	26.4	13104	42.7	90454	32.5
**Supervised treatment**														
Yes	35919	37.2	14181	40.7	1171	44.2	48989	44.2	2167	68.6	9513	31.0	111940	40.2
No	43741	45.3	16820	48.2	977	36.9	50715	45.8	770	24.4	9364	30.5	122387	43.9
Unknown	16947	17.5	3882	11.1	503	19.0	11011	9.9	221	7.0	11783	38.4	44347	15.9
**Outcome**														
Cure	73238	75.8	24654	70.7	1963	74.0	80357	72.6	2426	76.8	21567	70.3	204205	73.3
Default	7092	7.3	3657	10.5	199	7.5	9460	8.5	218	6.9	3124	10.2	23750	8.5
Death by TB	2896	3.0	1180	3.4	81	3.1	3623	3.3	89	2.8	1166	3.8	9035	3.2
Death, other causes	4582	4.7	1424	4.1	101	3.8	3994	3.6	75	2.4	1605	5.2	11781	4.2
Transferred	5320	5.5	2389	6.8	212	8.0	9097	8.2	249	7.9	1842	6.0	19109	6.9
MDR-TB	210	0.2	78	0.2	8	0.3	256	0.2	6	0.2	22	0.1	580	0.2
Unknown	3269	3.4	1501	4.3	87	3.3	3928	3.5	95	3.0	1334	4.4	10214	3.7
**Total**	**96607**	**100.0**	**34883**	**100.0**	**2651**	**100.0**	**110715**	**100.0**	**3158**	**100.0**	**30660**	**100.0**	**278674**	**100.0**

Thorax radiography assessments revealed high rates of results suggestive of TB among all colour/race categories, with a slightly higher rate in the black population (81%). Tuberculin tests were not performed in more than half (56.7%) of all notified TB cases, with the highest rates of non-compliance in the black and the 'mixed' populations (both 64.6%) (Table 3).

Pulmonary tuberculosis was reported in 82.3% of all cases, making it the most common clinical presentation of the disease among all colour/race categories. The highest rate of pulmonary tuberculosis was found in the indigenous population (86.6%). Extrapulmonary tuberculosis was predominantly reported in the white population (17%) compared to the other colour/race categories (Table 3).

In 33.4% of the notified cases, HIV testing did not take place. The lowest rates of HIV testing were found in the indigenous population (59.6%), while the highest rates of HIV testing were found in the white population (71.7%) (Table 3).

More than 40% of the control bacilloscopies (in the 2nd, 4th, and 6th months of treatment) did not take place. It is noteworthy that only 40.2% of cases received directly observed treatment (DOT), with a markedly higher rate of DOT among the indigenous population (68.6%).

‘Cured’ was the most common outcome for all colour/race categories. The highest cure rate was observed in the indigenous population (76.8%), and the lowest rate was observed in the black population (70.7%). Treatment default rates were also highest in the black population (10.5%) and lowest in the indigenous population (6.9%). Death by TB was most common in the black population (3.4%) and least common in the indigenous population (2.8%) (Table 3).

**Fig 2:**
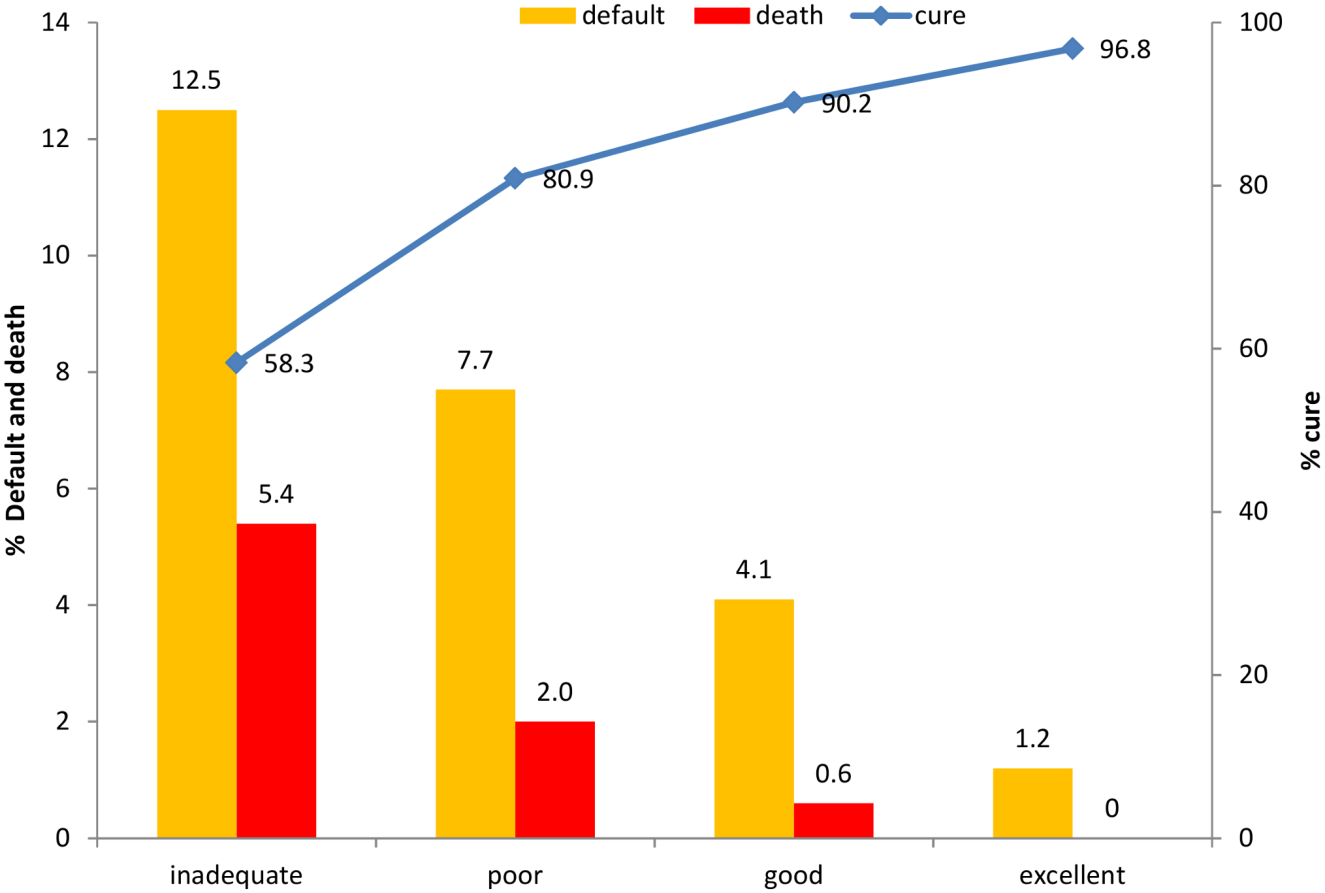
Relationship between treatment outcomes (on the left and right of the axis Y) and classification of the patient’s follow-up (on the axis X) for new cases of TB, Brazil 2008–2011. Note: On the left of the axis Y you can see the percentage of treatment defaults and death, while on the right of the axis Y you can see the percentage of cure. On the axis X could you see the status of the classification of the patient’s follow-up. According our data, rates of cure were lower (blue line), as well as rates of treatment default and death were higher (yellow and red column, respectively) in cases where the patient’s follow-up was inadequate. On the other hand, treatment outcome was better in the cases where patient’s follow-up were good and excellent (blue line).

Rates of cure were lower in cases where the follow-up was classified as inadequate (58.3%). High death rates and treatment default rates were remarkable in cases in which the follow-up was classified as inadequate (5.4% and 12.5%, respectively) in all colour/race categories (Fig 2).

It can be observed that treatment outcome is generally successful in cases with adequate follow-up. Better patient follow-up classifications resulted in higher cure rates (96.8) and lower rates of treatment default (Fig 2).

## Discussion

Our findings confirmed that although TB remains at an endemic level in Brazil, its geographical distribution has become homogenous among the macro-regions of the country in recent years. Marked ethnic and racial inequalities were observed regarding the clinical, epidemiological, and operational aspects of the disease, the follow-up of cases in treatment and treatment outcomes.

As described by other authors [21,22], our findings note higher incidences of TB in the North and Southeast regions of Brazil and lower incidences in the Central-West region. However, when the data are stratified by 'colour or race' in these regions, hitherto unknown incidence patterns surface—in particular, the higher burden of disease among indigenous and black people compared to other categories. It is worth noting that in this study, the Central-West region—where the number of TB notifications has traditionally been low in comparison with other regions—registered the highest incidence rates of TB in the indigenous population: more than 6 times higher than the national average.

In turn, high incidence rates of TB were found in the black population, reaching a peak of 93.3/100,000 in 2009, which exceeded the average incidence rate in the general population more than three times. The highest rates were detected in the South region, which has the best indicators of health and human development in the country [23]. Although the black population represents only approximately 4% of the population in the South region, 11.4% of TB case notifications during the period 2008–2011 occurred in this segment of the population in the South.

As was noted in a study by Chiavegatto-Filho and Laurenti [24], the inequalities in TB indicators between black patients and other patients can be explained by the more precarious living conditions among the black population, which include lower incomes and more limited access to health services.

The indigenous population’s vulnerability to TB bears similarities to the black population in the South region and can be explained similarly by poverty and limited access to health services as well as certain other characteristics of indigenous households. Generally, indigenous homes are small and crowded, with little natural light and ventilation. These characteristics contribute to the preservation of the TB bacillus in this environment and favour the spread of the disease [25].

Although approximately half of the notifications were concentrated in the age group 20–44 years in all colour/race categories, high morbidity rates in indigenous children were featured prominently in all regions. The rates of TB among indigenous children were substantially higher than the 5% expected by the PNCT among children up to 15 years in the general population of Brazil [8]. Cases of TB in children under 10 years of age, independent of colour or race, can be considered as an indicator for active transmission of TB in the community due to their contact with bacilliferous adults, and are a sign of failed contact surveillance, especially in indigenous villages located in the remote hinterland areas of Brazil [26].

From this perspective, the high rates of TB case notification among the indigenous population living in rural areas are startling [27]. Various studies based on primary data from the indigenous communities have revealed high TB incidence rates [15,16,28–30], particularly among children and adolescents [16,26,29,31], prevalence rates of latent TB infection surpassing 40% [32], and patterns of recent transmission in some indigenous villages in Brazil, supporting our hypothesis of continuous TB transmission in these locations [26,33].

In addition, some authors have proposed theories that these high incidence rates could be associated with genetic polymorphisms inducing an inefficient immune cell response that would direct the course of the infection and leave the indigenous population more vulnerable to the disease [34,35]. However, despite such theories, there is strong evidence that it is the situation of extreme poverty in which these populations live—where hunger, a permanent state of food insecurity, unemployment, and low income or no source of income are coupled with a high prevalence of malnutrition, anaemia, and intestinal parasites—that contributes to the continued high TB burden in the Indigenous Territories [13,27,36–40].

The highest rates of illiteracy were also found among the indigenous TB patients, followed by black patients. According to San-Pedro and Oliveira [41], these low educational levels are closely associated with the precarious socioeconomic conditions that further increase their vulnerability to TB infections as well as a host of other diseases related to poverty. A likely explanation for this disparity is that, for the indigenous population, access to formal education is still difficult, particularly in the North region of Brazil. However, the variable ‘education’ in the SINAN database is often omitted or only partially completed, which obstructs further analysis of this theme.

A higher number of negative results in the bacilloscopies of the 1st and 2nd sputum samples was observed in the indigenous population, which could be partly explained by inadequate orientation of the patient at the time of sample collection or—as proposed by recent studies—by inappropriate storage and transport of the samples and technical incompetence resulting in incorrect reading of the sputum smears [25,28,42].

According to the 'III Diretrizes para TB' by the SBPT, sputum culture is recommended for suspected cases of TB that present a negative bacilloscopy and is considered the most accurate diagnostic tool for TB [20]. Unfortunately, our analysis has shown that sputum culture is underused in practically the entire country. Given this finding, it was noteworthy that sputum culture was employed at the highest rate in the indigenous patient population, in contrast to the examination levels observed in the other colour/race categories.

It is also noteworthy that sputum culture occurred most frequently in the Central-West region of the country. According to recent studies, [24,26], the laboratory services for the diagnosis of TB in the indigenous population in the state of Mato Grosso do Sul, which is home to the 2nd-largest indigenous population contingent in Brazil, have been improving for at least a decade. This could partly explain our findings.

The predominance of thorax radiography over sputum bacilloscopy as a diagnostic examination for TB was also observed in all colour/race categories. These findings can probably be explained by its low cost, the availability of radiography equipment in the healthcare units, and the relative ease of performing this examination. When radiography is well indicated in diagnostic investigations of respiratory symptoms, the results can produce valuable clinical information [43].

Low rates of tuberculin tests were also found in all notified cases in the country. However, in cases where tuberculin tests were used, high rates of skin reactions above 10 mm were registered among indigenous patients. As has been proposed by other authors [28,34], this fact supports a scenario of high prevalence rates of *Mycobacterium tuberculosis* infection in this population.

Despite the recommendations by the World Health Organization (WHO) that all cases diagnosed with TB should be offered an HIV test [8], we found important inequalities in the access to this test among the colour/race categories. Most prominently, the lowest rates of HIV testing were found among the indigenous population, while the highest rates of HIV testing were found among the white population (28.3%). In a recent study, Basta et al. [16] identified a similar pattern of non-compliance with the recommendations for HIV testing in the indigenous population in Mato Grosso do Sul. HIV tests were not performed for approximately half of all notified TB cases among the indigenous population. As a result, the healthcare service misses out on the opportunity to detect, manage, and control TB/HIV coinfection in this population.

Performing control bacilloscopies in the 2nd, 4th, and 6th months of treatment is considered fundamental for successful follow-up of TB cases. However, our analysis showed that in more than 50% of the cases, there were no control test registrations. According to a study by Belo et al. [15], in municipalities in the Amazon region along the northern arch of Brazil's international borders, the cases where bacilloscopy was not performed or performed only once presented a 12-fold increased risk of treatment default compared to cases where two or more bacilloscopies were performed during the treatment schedule. These authors argue that performing control bacilloscopies is a potential way of creating a bond between the patients and the healthcare service, and thus, such procedures contribute to reducing the risk of treatment default.

In accordance with WHO recommendations, all cases of TB must receive directly observed treatment (DOT) [8]. However, our data reveal that DOT failed to reach 100% of cases in all colour/race categories. Despite this failure, the high rates of DOT among the indigenous population (in contrast to the black population, which presented the lowest rates) indicate that—at least in this respect—the healthcare service organizations have achieved a remarkable level of follow-up among the indigenous population. However, it is of concern that no specific policy has been directed at the black population thus far, despite this population’s high vulnerability to TB [44].

The analysis of the outcomes of notified TB cases showed that no colour/race category reached the objectives for cure established by the PNCT [8]. Similar to findings from other studies [15,16], the highest cure rates were registered among the indigenous patients. We observed the lowest cure rates (70.7%) and the highest rates of treatment default (10.5%) and death by TB (3.4%) among the black population. These findings demonstrate that there is great disparity in treatment follow-up between the colour/race categories and call for additional efforts to reach the goals agreed on with the WHO, especially regarding TB control in populations who live in vulnerable situations.

Although exclusively empirical, the classification of the follow-up system for patients employed in this study is congruent with the evaluation logic of the PNCT, showing that treatment success is directly linked to the structuring and surveillance capacity of the healthcare service. Patients who undergo supervised treatment and are duly followed up with during treatment have higher rates of cure, lower rates of default, and lower frequencies of complications and death.

Even though this study yielded multiple findings, the following limitations must be mentioned. Studies based on secondary data are subject to under-registration of cases, classification and/or diagnostic errors, and low representativeness of specific population segments. Moreover, analyses are limited to the variables as they are structured in the information systems [45,46].

Although we have shown that important improvements have occurred in completing entries for the variable 'colour or race' in recent years, gaps still exist in completing entries for some other variables in the SINAN. In certain situations, it is possible that there has been systematic error in acquiring complete entries for variables in the SINAN, which would mean that the estimates are distorted. Theoretically, such errors compromise the identification of the true disease profile of TB in Brazil [45,47]. However, there is no reason to believe that possible classification errors have affected the registration of the variable 'colour or race'. According to a recent document published by the Brazilian Ministry of Health [14], reasonable progress has been made in completing entries for the variable 'colour or race' in national health information systems. Moreover, the data make it possible to undertake analyses that aim to elucidate inequalities in indicators for various diseases and deaths in recent times. In this respect, we think that the analyses presented in this study are useful for demonstrating inequalities in the distribution of TB across categories of 'colour or race', both among and within the macro-regions of Brazil.

Another point to consider is that in the colour/race category, 'indigenous' covers the entire ethnic and cultural diversity of the indigenous peoples of Brazil. According to the last national census (2010), this is one of the most ethnically and culturally diverse populations on the planet; it includes more than 300 ethnic groups speaking more than 200 different languages [48]. The current categories available in the registration of disease and death in Brazil conceal this diversity. At least, regarding TB, it is known that the distribution of the disease is not homogenous among the indigenous peoples because concentrations of cases exist in some groups in the Amazon Basin and in the Central-West region of the country.

Considering the limitations outlined, the results presented here should be interpreted with caution. In particular, it is important to not speculate that the observed differences in TB disease indicators could be attributed to problems of a genetic or biological nature [49]. As in other parts of the world, the concept of ethnicity in Brazil is the product of complex historical and social constructions. From this perspective, the racial differences observed in the indicators for health seem to be more associated with socioeconomic inequalities and access to healthcare and educational services than with genetic risk markers or other biological parameters [50,51].

## Conclusions

Our findings revealed, in an unprecedented manner, important ethnic and racial inequalities in the notification of cases of TB regarding both the follow-up of cases in treatment as well as the clinical and epidemiological parameters, with emphasis on the higher incidence rates among the indigenous and black populations.

Finally, it is important to consider that the control of TB in Brazil is a problem neither limited to the healthcare sector nor restricted to its actions and services. It is of vital importance for the PNCT to involve other government bodies in the design, implementation, and development of control strategies and to encourage social development initiatives to improve the general living conditions and health of the areas and specific segments of the population where TB is an important public health problem.
